# Leveraging data visualization to improve the use of data for global health decision-making

**DOI:** 10.7189/jogh.09.020319

**Published:** 2019-12

**Authors:** Tricia Aung, Debora Niyeha, Rebecca Heidkamp

**Affiliations:** 1 Department of International Health, Johns Hopkins University Bloomberg School of Public Health, Baltimore, Maryland, USA; 2 Institute for International Programs, Johns Hopkins University, Dar es Salaam, United Republic of Tanzania

The surge of available global health data necessitates developing creative approaches to communicate evidence to decision-makers. This “data revolution” reflects both increased demand for data to assess programs and policies and improved technologies to rapidly collect and disseminate data [1]. More available data does not necessarily translate into more evidence-based program and policies in global health. There is limited understanding of how to effectively promote use of data for decision-making [2].

Data visualization – the visual representation of data – techniques are increasingly being used in global health. The DHS Program’s STATcompiler (https://www.statcompiler.com/en/) and UNICEF’s Data portal (https://data.unicef.org/) are data exploratory tools that allow users to visualize household survey data collected by these institutions. Dashboards are used by governments in low- and middle-income (LMICs) countries and development partners to present indicators of interest. Dashboards exist for every global health focus area, and sometimes numerous dashboards exist that visualize the same topic. Scorecards, another data visualization technique, are also widely used in global health. For example, the Government of Tanzania has used reproductive, maternal newborn child health (RMNCH) scorecards to track progress towards achieving maternal and child health intervention targets since 2014.

This viewpoint describes efforts undertaken by the National Evaluation Platform (NEP) project to strengthen capacity for analysis and communication of RMNCH & nutrition (RMNCH&N) data to inform policy and program planning decisions by governments in four sub-Saharan African countries between 2013-2018. Specifically, this viewpoint aims to describe the evolution of efforts to incorporate data visualization and its impact on communicating actionable key messages to decision makers

## DATA VISUALIZATION TRAINING FOR NEP COUNTRY TEAMS

The Institute for International Programs at Johns Hopkins University (IIP-JHU) collaborated with government institutions to develop NEPs in Malawi, Mali, Mozambique, and Tanzania. The NEP approach brought together representatives from national statistical offices, government ministries, and public research institutions for training and mentorship activities to systematically identify and answer priority RMNCH&N program and policy questions with diverse data sources [3]. Even though participants worked with RMNCH&N data as part of their professional roles, we found that baseline data literacy skills – including identifying and communicating key messages and producing graphs in Excel – were highly variable and generally low [4]. An external midterm evaluation across the four NEP countries found that that the project had overestimated the baseline technical capacity of NEP country team members. The evaluators recommended increased focus on building communication capacity to support translation of findings into policy recommendations.

In response to feedback from the external evaluation, during the second half of the NEP project, the IIP-JHU team developed a suite of communication training resources including data visualization curriculum that emphasized how to choose an appropriate data visualization to communicate specific key messages. The curriculum reflected best practices rooted in cognition research and hands-on exercises to produce figures using Excel and Stats Report (https://statsreport.io/) [5]. The project incorporated the curriculum in workshops with all four country teams and carried out enhanced activities in Tanzania.

The external final evaluation of the NEP cited data visualization and presentation as an important area of capacity improvement through in-depth interviews with team members and their supervisors:

“I have the skills to decide which methodology and visualization I can use, and people can understand what I’m presenting to them.” *– NEP team member*

“I was impressed with these young people that were presenting. They had grown substantially from the previous meetings.” *– supervisor of NEP team member*

## EXPLORING DATA VISUALIZATION PREFERENCES AND INTERPRETATION CAPACITY IN TANZANIA

While developing the data visualization curriculum for NEP, it became clear that there is limited data visualization research within low-resource settings, despite the increasing popularity of data visualization in global health. Existing data visualization research focuses on how people perceive and interact with standard and novel visual representations of data. This research is predominately conducted by the fields of computer science, engineering, and information science. Modern data visualization studies frequently recruit subjects virtually through Amazon Mechanical Turk (https://www.mturk.com/); a majority of subjects are in the US, younger, and have at least a bachelor’s degree [6,7].

In August 2017, the NEP Tanzania team conducted a study to characterize data visualization preferences and interpretation capacity among 25 public-sector RMNCH&N decision-makers working at national level in Tanzania [8]. The team probed participants about their background in statistics and had them complete a series of exercises to describe key messages and rank different data visualization approaches using Tanzania RMNCH&N data. The study revealed wide variability in basic data literacy and statistical skills across participants. All participants identified data as a critical aspect to their professional role, but very few had received any statistics or data use training since university. Many participants struggled with identifying key messages in figures and overwhelmingly preferred bar graphs and pie charts over alternative visualization approaches more supported by data visualization best practices and/or used increasingly in global health. Participants also recommended improving figures by writing key messages adjacent to figures, ensuring figures can be understood by those colorblind, and avoiding including multiple indicators within a single graph. This study highlights how we cannot assume that decision-makers are able to interpret figures even if evidence presented is seemingly compelling. These findings further emphasize the importance of using data visualization approaches that are comprehensible to target users and the need to more actively bridge gaps in data literacy.

## APPLYING DATA VISUALIZATION TO DISSEMINATE EVALUATION FINDINGS IN TANZANIA

The results of the Tanzania data visualization study influenced how the team disseminated results from their evaluation and there is some evidence that this approach was effective. For example, despite study participants’ discomfort with stacked bar charts and dot plots, the team still incorporated these types of graphs but provided supplemental guidance on how to present these types of graphs. During dissemination events, the team carefully described how to interpret each graph and emphasized the key message. In their presentation, the NEP team visualized the insufficient number of skilled health workers to provide Comprehensive Emergency Obstetric and Newborn Care (CEmONC) using a lollipop plot – an alternative to a bar chart. Immediately following a dissemination event the Minister of State from the President Office – Regional Administration and Local Government (PO-RALG) endorsed all NEP recommendations for action and requested a decree for two new doctors in each facility being built or upgraded to provide CEmONC services.

**Figure:**
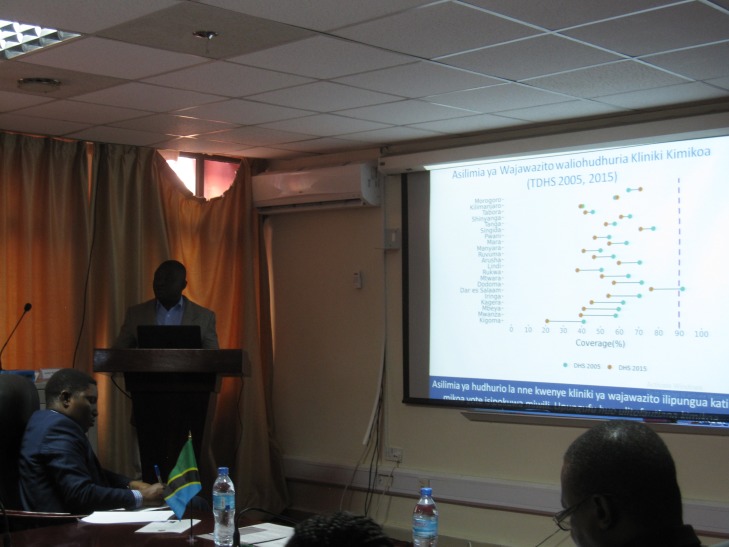
Photo: National Evaluation Platform Tanzania presentation of a dumbbell plot visualizing antenatal care coverage by region to the Tanzania President Office – Regional Administration and Local Government (from Tricia Aung’s own collection, used with permission).

## ESTABLISHING A DATA VISUALIZATION HUB WITHIN THE TANZANIA NATIONAL BUREAU OF STATISTICS

In response to interest sparked by dissemination of study findings, the NEP Tanzania team led by the National Bureau of Statistics held four trainings on basic data literacy and data visualization with different RMNCH&N government institutions in Tanzania in the final months of the project. The team trained over 70 individuals through four day-long trainings with staff from the National Bureau of Statistics, the Ministry of Health Reproductive and Child Health Section, PO-RALG, and Tanzania Food and Nutrition Centre. The National Bureau of Statistics, who led NEP Tanzania, is able to lead future trainings and support trainees with data interpretation and visualization.

## CONCLUSION

Incorporating data visualization techniques and basic data literacy training should be part of any statistical capacity building efforts and an intentional approach towards evidence-based programs and policies. Sustainable Development Goal (SDG) 17 prioritizes strengthening national statistical systems by ensuring countries have national statistical legislation and sufficient funding. However, strong institutional commitment to collecting statistics does not guarantee that decision-makers and the public can use and understand data produced.

The NEP experience highlights the importance of integrating data visualization techniques and basic data literacy training with overall statistical capacity efforts. There is need to expand data visualization research and create data visualization training opportunities in LMICs. Positioning national statistical bureaus that are responsible for large-scale cross-sector data collection in LMICs as data visualization experts could help improve the use of health, agriculture, and trade data, as well as data from other sectors. Engaging local universities and research institutes may encourage development of more locally driven data visualization solutions.
